# Experimental Demonstration of Electromagnetically Induced Transparency in a Conductively Coupled Flexible Metamaterial with Cheap Aluminum Foil

**DOI:** 10.1186/s11671-019-3180-y

**Published:** 2019-12-02

**Authors:** Jie Hu, Tingting Lang, Weihang Xu, Jianjun Liu, Zhi Hong

**Affiliations:** 1 Institute of Optoelectronic Technology, China Jiliang University, Hangzhou, 310018 China; 2 Centre for THz Research, China Jiliang University, Hangzhou, 310018 China

**Keywords:** Electromagnetically induced transparency, Terahertz, Metamaterial, Surface currents, Potential difference

## Abstract

We propose a conductively coupled terahertz metallic metamaterial exhibiting analog of electromagnetically induced transparency (EIT), in which the bright and dark mode antennae interact via surface currents rather than near-field coupling. Aluminum foil, which is very cheap and often used in food package, is used to fabricate our metamaterials. Thus, our metamaterials are also flexible metamaterials. In our design, aluminum bar resonators and aluminum split ring resonators (SRRs) are connected (rather than separated) in the form of a fork-shaped structure. We conduct a numerical simulation and an experiment to analyze the mechanism of the proposed metamaterial. The surface current due to LSP resonance (bright mode) flows along different paths, and a potential difference is generated at the split gaps of the SRRs. Thus, an LC resonance (dark mode) is induced, and the bright mode is suppressed, resulting in EIT. The EIT-like phenomenon exhibited by the metamaterial is induced by surface conducting currents, which may provide new ideas for the design of EIT metamaterials. Moreover, the process of fabricating microstructures on flexible substrates can provide a reference for producing flexible microstructures in the future.

## Introduction

Metamaterials [1, 2] are artificially engineered composites with subwavelength structures. Their physical properties, such as the dielectric constant, permeability, and conductivity, can be arbitrarily designed by changing the structure and size of the periodic lattice Therefore, many interesting phenomena can be realized by tailoring the geometry of the unit cells, with immense application potential such as metalenses and related wavefront regulation in metasurface [3–8], negative index media [9, 10], polarizers [11, 12], metamaterial absorbers [13–15], and reconfigurable metadevices [16]. The combination of metamaterials and two-dimensional materials further widens the research scope [17–19]. Among them, the analog of electromagnetically induced transparency (EIT) exhibited by metamaterials is a research hotspot.

EIT [20] is a quantum-mechanical phenomenon originally observed in atomic or molecular systems based on destructive interference between transitions driven by two laser beams. EIT renders a highly opaque medium, transparent over a narrow spectral region because of the lack of absorption, which is now realized in waveguide structures [21, 22]. On the other hand, the analog of EIT is also observed in metamaterials, characterized by a relatively narrow transparent peak in a wide opaque region in the spectrum. Various resonances can occur in metamaterials because of the interaction between the periodic structures and the incident electromagnetic field. Furthermore, the destructive interference between the different resonances causes EIT-like phenomena in metamaterials. Many researchers are now engaged in this topic, and a variety of structures has been proposed to realize this phenomenon. The current common EIT-forming mechanism is based on the destructive interference between “bright modes” and “dark modes”. For example, the inductive-capacitive (LC) oscillation in metallic split ring resonators (SRRs) suppress the localized surface plasmon (LSP) resonance in metallic bars [23–26]; quadrupole suppress dipole wherein metamaterials combined with bar-shaped resonators [27–30] or bar-shaped grooves [31, 32] in different directions; the magnetic resonance in a dielectric block or a dielectric ring resonator suppresses the electrical resonance in a dielectric bar resonator [33–35]. The destructive interference between a bright mode with a lower quality factor (Q factor) and a bright mode with a higher Q factor (also known as a quasi-dark mode) also induces analog of EIT in metamaterials [36]. For instance, LC resonances with a higher Q factor in SRRs suppress the LSP resonances with a lower Q factor in metallic ring resonators [37–39]; a guide mode with a higher Q factor in a waveguide layer suppresses the resonances in a periodic structure above the waveguide layer [40–42]. Some scholars incorporated controllable substances in the design to realize all-optical tuning of EIT [43, 44] or active electronic controlling of EIT [45, 46]. In most designs, particularly in metallic metamaterials, antennae with different modes are always separated; they interact with each other through near-field coupling.

A metal has a high electrical conductivity in the terahertz band. Moreover, a metallic metamaterial structure is subjected to surface plasmon when the resonance is excited, and a surface conduction current is induced at the same time, which makes conductively coupling possible [47–49]. Here, we propose a design wherein different resonators interact via surface currents. We propose a conductively coupled terahertz metallic metamaterial, in which the bright and dark mode antennae are connected in the form of a fork-shaped structure to realize analog of EIT.

## Methods/Experimental

**Fig. 1:**
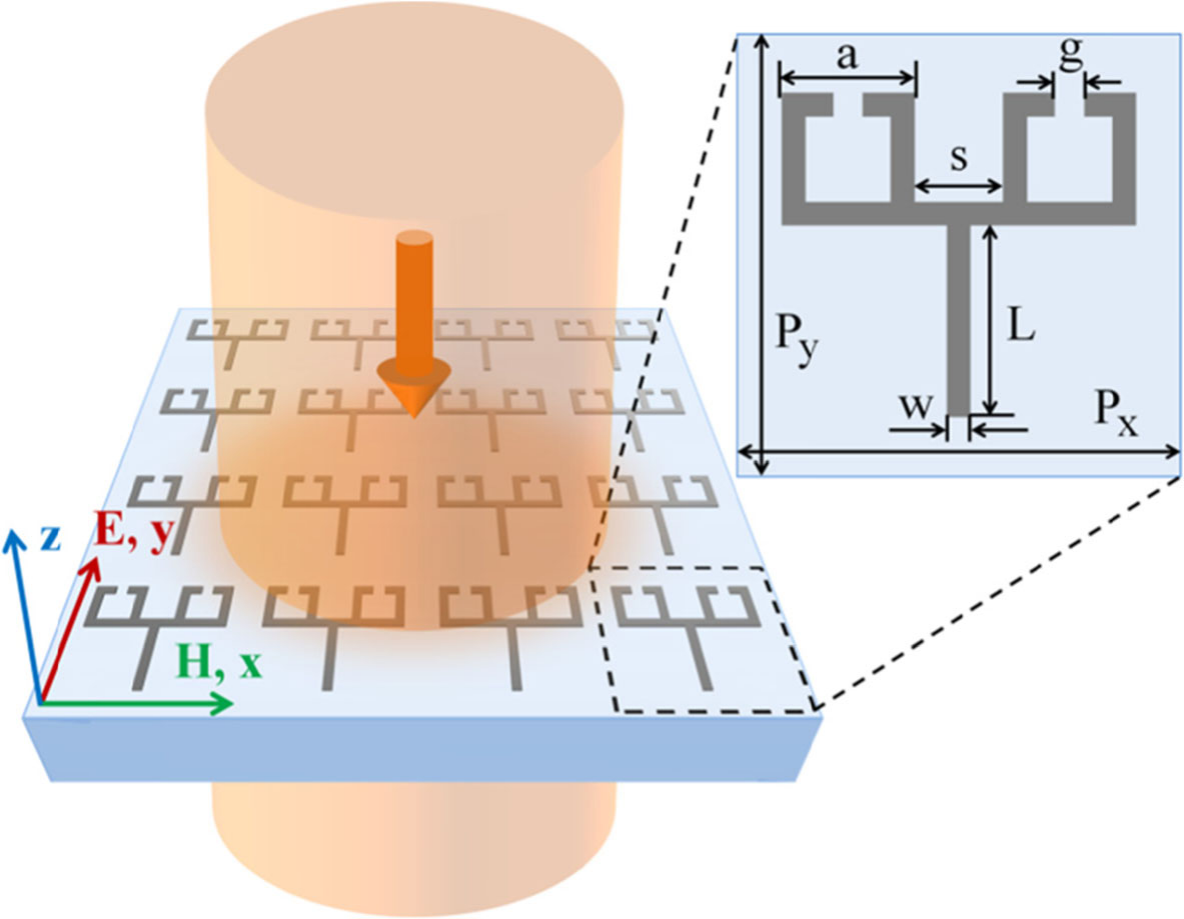
Schematic of the conductively coupled terahertz EIT metamaterial

Figure 1 shows the design of the proposed conductively coupled terahertz metamaterial. The structure is a fork-shaped periodic array formed by interconnecting aluminum bar resonators and aluminum SRRs.

The periods of the unit cells are equal in both the *x* and *y* directions; *P*_*x*_ = *P*_*y*_ = 150 μm. The length of the square SRR is *a* = 45 μm. The gap between the two SRRs is *S* = 30 μm. The slit gap of the SRR is *g* = 10 μm. The length of the aluminum bar is *L* = 65 μm. The line width of the aluminum strips and SRRs is *w* = 8 μm. The substrate is made of polyethylene terephthalate (PET). For all the simulations, corresponding full-wave simulations were carried out using CST Microwave Studios (the metal selected was aluminum with a conductivity of 3.56 × 10^7^ S/m, and the permittivity of the PET substrate is 3.2). The thickness of the aluminum structure was set to 150 nm in the simulation. We assumed the incident light to be a plane wave propagating in the opposite direction of the *z*-axis. The electric and magnetic fields of the incident light are polarized along the *y*- and *x*-axes, respectively.

As for the experiment, we used the purchased composite of a PET-aluminum film as the raw material. This kind of commercial aluminum foil is very cheap and often used in common food package. Lithography (laser direct writing) and wet etching processes were employed in fabrication. Compared with conventional micro/nanofabrication technologies, laser direct writing technique offers several distinct advantages, such as designable processing without the use of masks, ease of integration with given devices, and feasibility of 3D structuring capability [50]. As the PET substrate is very soft owing to its thickness of approximately 20 μm, we first added some volatile liquid on a flat and clean quartz substrate, then flattened the PET–aluminum film composite on the quartz substrate, and discharged the air between the composite material and the quartz substrate. After the liquid evaporates, the flat composite firmly attaches onto the surface of the quartz substrate; this is convenient for the subsequent spin-on photoresist and photolithography processes.

**Fig. 2:**
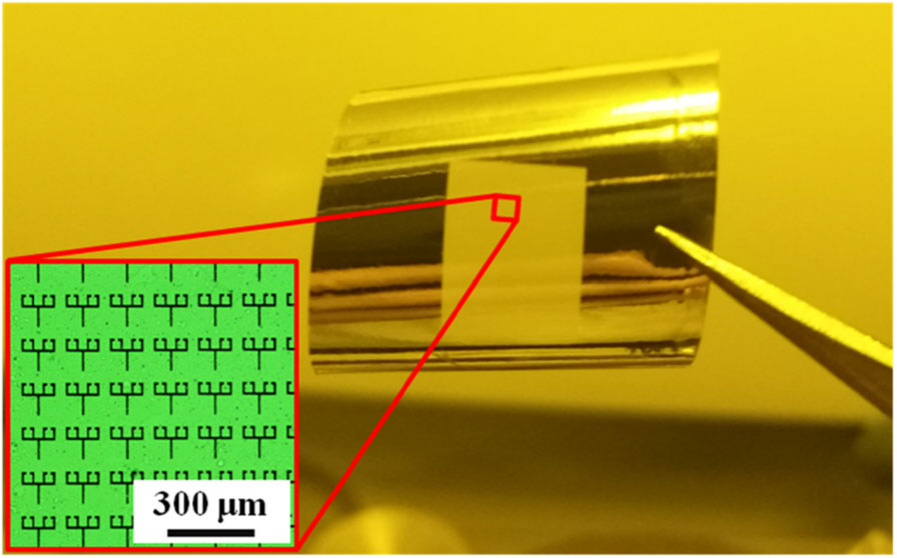
Fabricated sample of the conductively coupled terahertz EIT metamaterial. The microscopic image of the fabricated conductively coupled structure is shown in the inset

After the fabrication of the metamaterial, it was gently removed from the quartz substrate for the following testing. Terahertz time domain spectroscopy (THz-TDS) was then employed to measure the complex transmission coefficients of the samples at normal incidence for *y*-polarization incidence. The flexible material, shown in Fig. 2, is the fabricated metamaterial sample, in which the seemingly transparent intermediate portion is a 60 × 80 periodic array. The microscopic image of the fabricated conductively coupled structure is also shown in the inset. The above method provides a reference for fabricating microstructures on a flexible material to realize a flexible device.

## Results and Discussion

**Fig. 3:**
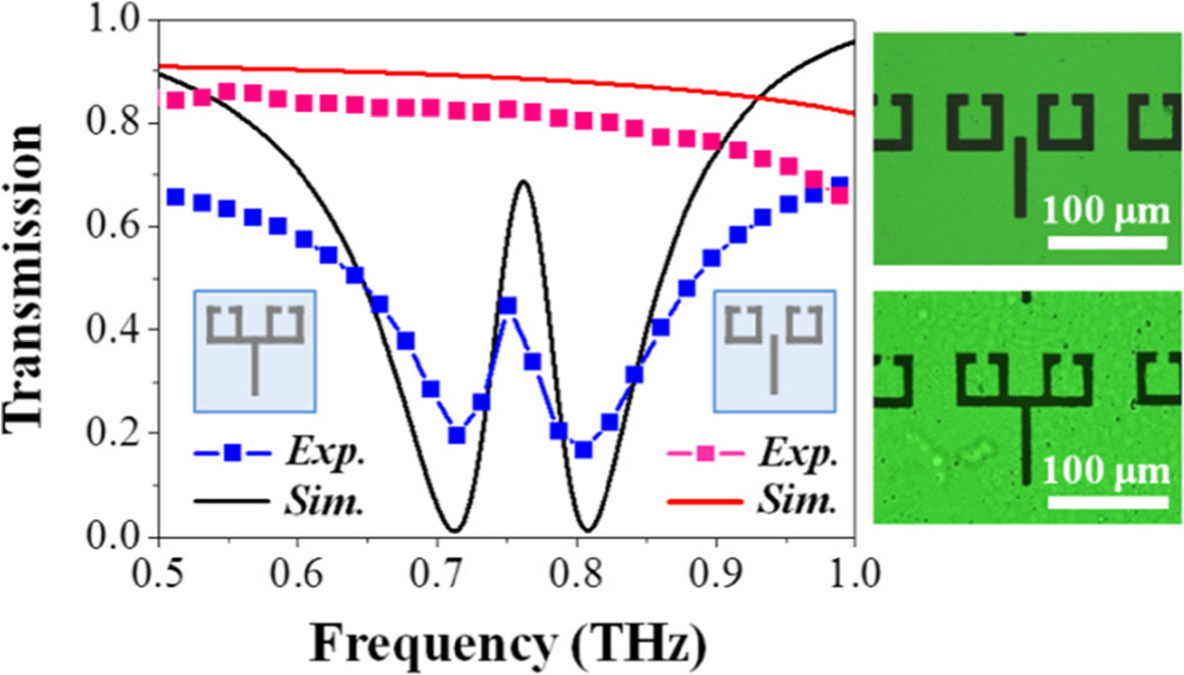
Simulated and measured spectra of the conductively coupled terahertz metamaterial and those of the conventional one wherein the bar resonator is separated from the SRRs. The microscopic images of the corresponding structures are also shown next to it

Figure 3 shows the simulated and measured frequency spectra of the conductively coupled terahertz metallic metamaterial, indicated using a black solid line and a blue dotted line, respectively. A microscopic image of the structure is also shown next to it. The measured curve and the simulation result are in good agreement. The fabricated metamaterial exhibits a transmission peak at approximately 0.76 THz. The measured EIT peak is in the range of approximately 0.15–0.45, which is lower than that determined from the simulation (0.7). According to the ratio of the transmission peak’s central frequency to the full width at half maximum (FWHM), the Q-factor of simulated spectrum is 17.5, which declines to approximately 12 in experimental result due to the loss and measurement accuracy. On the other hand, to compare the conductively coupled terahertz metamaterial with conventional structures wherein the metallic bar resonator and metallic SRRs interact via near-field coupling, we fabricated and tested a sample wherein the bar resonator is separated from the SRRs. Figure 3 shows the simulated and measured frequency spectra of the conventional structure as well, indicated using a red solid line and a pink-dotted line, respectively. For the conventional separated structure, neither EIT phenomenon nor resonance occurs in the frequency range of 0.5–1 THz. By comparison, we find that the mechanism of our conductive EIT metamaterial is different from that of the conventional separated structure.

**Fig. 4:**
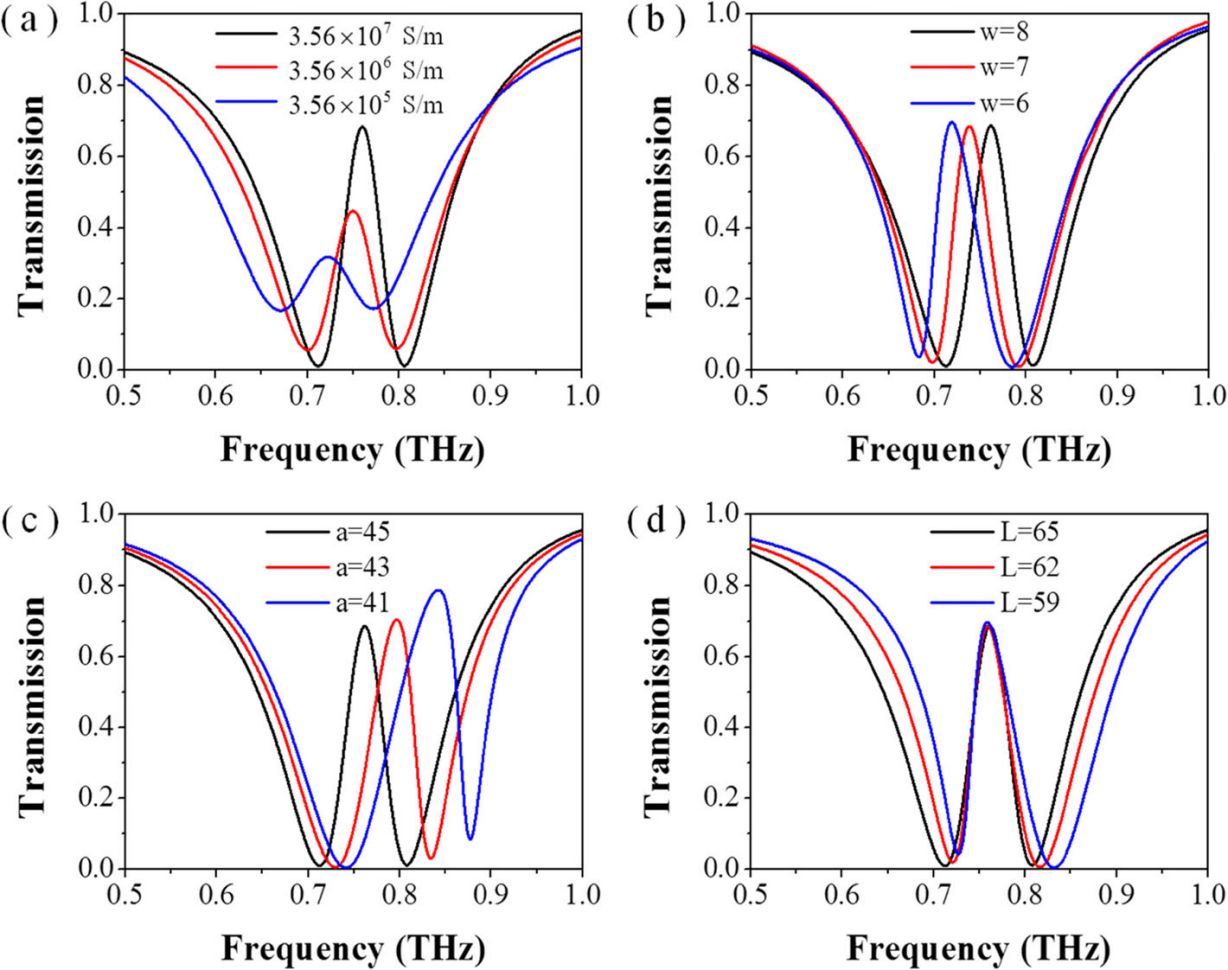
Simulated spectra of the conductively coupled terahertz metamaterial with various structural parameters of **a** conductivity of aluminum; **b** the line width of the aluminum strips and SRRs; **c** the length of the square SRR; **d** the length of the aluminum bar

Although the experimental results mostly agree with the simulation results, there are some minor differences. We analyzed and simulated the effects of different parameters on the results, as shown in Fig. 4.

First of all, metamaterial structure is composed of aluminum. It is well known that the metal aluminum surface is prone to form a dense oxide film, which leads to the reduction of the conductivity of the structure and weakens the conductively coupling effect of the structure. The effect of conductivity on metamaterial EIT phenomenon is shown in Fig. 4a. As the conductivity decreases (from 3.56 × 10^7^ S/m to 3.56 × 10^5^ S/m), the EIT amplitude decreases significantly, and the frequency shifts slightly, from 0.76 to 0.72 THz. In addition, the size of the fabricated metamaterials has also been measured with a microscope. It is found that there are some differences between the size of the fabricated structure and the parameter setting in the simulation process. Here, we list some obvious difference: the line width of the aluminum strips and SRRs, *w*, (6.5~7.5 μm) is thinner than the designed value (8 μm), and the length of the square SRR, *a*, (43~41 μm) is smaller than the designed value (45 μm), the length of the aluminum bar, *L*, (61~62 μm) is shorter than designed value (65 μm). The influences of *w*, *a*, and *L* on EIT effect are shown in Fig. 4b, c respectively. As shown in the Fig. 4b, as the *w* decreases, the frequency of the EIT phenomenon decreases. Since the parameter *w* involves both the SSRs and the metal bar structure, the change of this parameter causes the shift of the absorption frequency and the transmission frequency of the EIT. While in Fig. 4c, d, as the *a* and *L* decrease, the transmission peak and the absorption range of the EIT phenomenon appear blue shift respectively, that is, the frequency increases. The combination of all these differences in experiment and simulation ultimately led to the difference between the actual measured spectrum and the simulated spectrum. What’s more, according to the frequency shift of the absorption region and the transmission peak caused by the variation of parameters in Fig. 4, it can also be concluded that although the bright and dark mode antennas are integrated in the structure, there are also strict requirements for the size of both antennas to make these two frequencies of modes matched with each other.

**Fig. 5:**
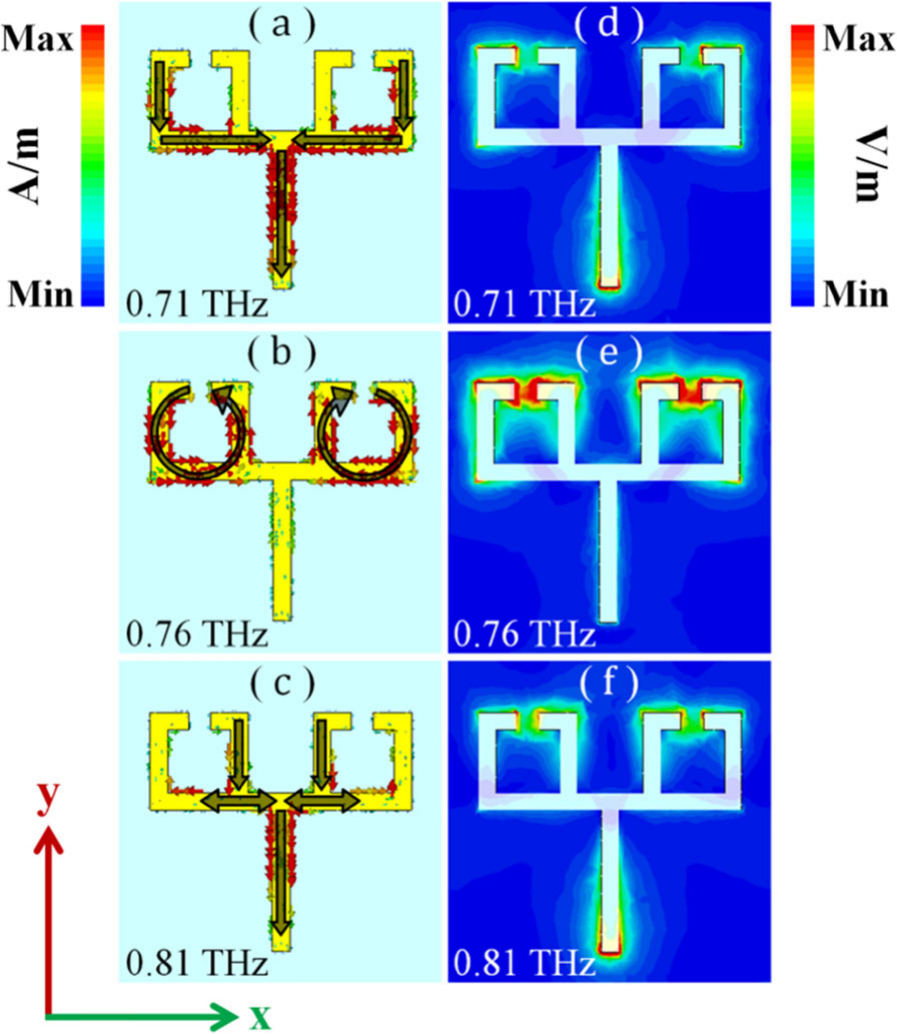
Surface current at different frequencies: **a** EIT peak frequency, **b** transmission dip with lower frequency, **c** transmission dip with higher frequency. Electric field distribution at different frequencies: **d** EIT peak frequency, **e** electric field distribution at the transmission dip with lower frequency. **f** Electric field distribution at the transmission dip with higher frequency

To further analyze the EIT-forming mechanism of the conductive metamaterial, we simulated a surface current and an electric field distribution at the EIT peak frequency (0.76 THz) and at the transmission dips (0.71 and 0.81 THz), as shown on the left and right sides of Fig. 5, respectively. As shown in Fig. 5a, the surface current flows from the outer metallic arm of the SRRs to the bar resonator. This is consistent with the polarization direction of the incident electric field, i.e., from one end to the other end along the *y*-axis with back-and-forth oscillation, thus exhibiting a typical LSP resonance.

Figure 5b shows the surface current distribution at the EIT frequency (0.76 THz). The vortex surface current is mainly concentrated at the SRRs, indicating a fundamental LC resonance and LSP resonance suppression. As for the second transmission dip at a higher frequency (0.81 THz), the surface current distribution is from one end to the other end along the *y*-axis direction, indicative of an LSP resonance, as shown in Fig. 5c. However, the surface current flows through the inner metallic arm of the SRRs. In comparison with the path, shown in Fig. 5a, the conduction path of the surface current, shown in Fig. 5c, is shorter, which corresponds to a shorter resonance wavelength and a higher resonance frequency. Figure 5e, d, and f show the electric field distributions at the frequencies of the EIT transmission peak and two transmission dips besides the EIT peak. In Fig. 5e, the electric field energy is mainly concentrated at the gaps of the SRRs, whereas in Fig. 5d and f, the electric field energy is mainly concentrated at the two ends of the structure. These phenomena correspond to their respective surface current distributions.

**Fig. 6:**
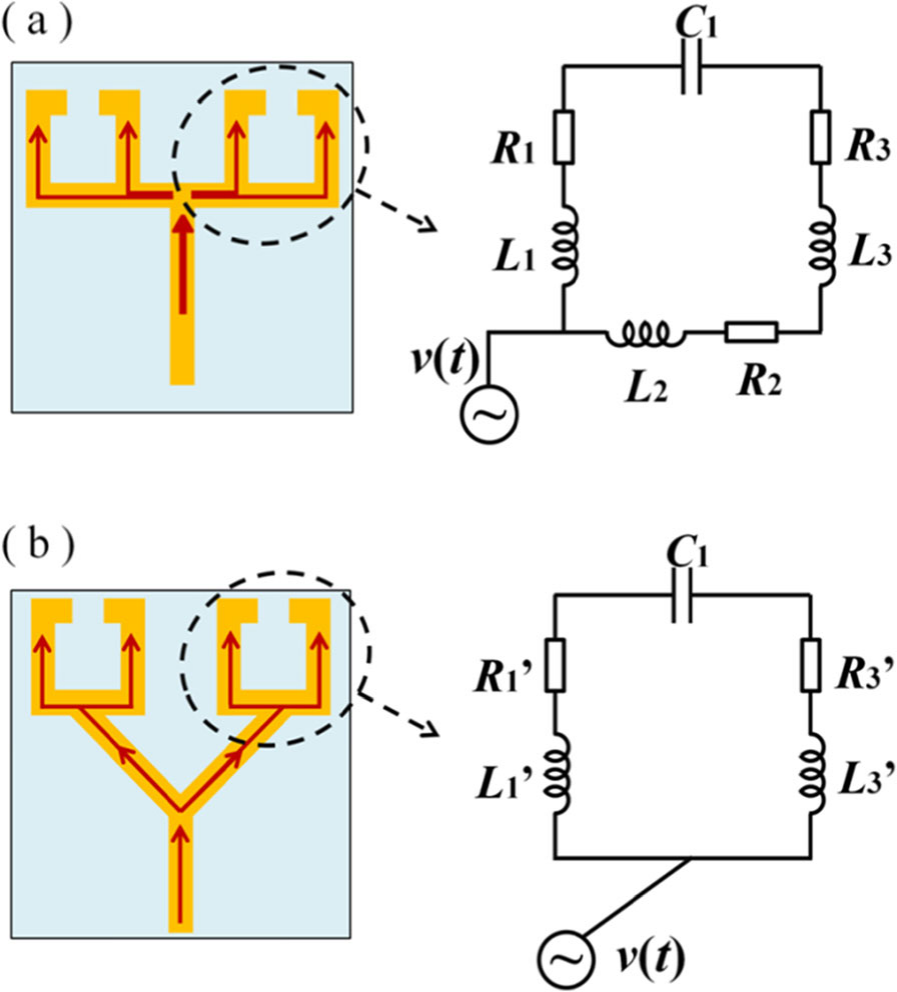
Electric circuit modeling the response of the conductively coupled terahertz metamaterial in which the junctions are located **a** on one side of the vertical center line of the SRRs; **b** on the vertical center line of the SRRs

In fact, the generation of this LC resonance (dark mode) can also be explained from the knowledge of the circuit. When the LSP resonance (bright mode) is excited, the surface current oscillates back and forth along the *y*-axis. When the current flows to the point connecting the bar resonator and the SRRs, there is a bifurcation in the conduction path. The current flows from the junction to the split gaps of the SRRs through two conductive paths. One of the paths is along the metallic arm outside the SRRs, consistent with the flow direction of the surface current shown in Fig. 5a. The other one is via the metallic arm inside the SRRs, as shown in Fig. 5c. Here, this phenomenon can be analogized to the process of charging and discharging the slits of the SRRs. In fact, there already have been literatures modeling the coupled resonator of metallic bar and SRRs as RLC circuit [23], and the concept of the “LC resonance” has been utilized for many years [45, 51]. The slit of the metallic SRR can be regarded as a capacitor. When the surface current is conducted on the metal arm, although the conductivity of the metal is high, some resistance still exists. Moreover, under the high-frequency oscillation of electromagnetic waves, there is a certain hindrance to the high-speed change of the surface current. That is, there is an inductance. The resistance and inductance of the metal arm are proportional to the length of the metal arm. If the two paths on the outer side and the inner side after the bifurcation are asymmetrical, as shown in Fig. 6a, *R*_*1*_ is smaller than the sum of *R*_*2*_ and *R*_*3*_, and *L*_*1*_ is smaller than the sum of *L*_*2*_ and *L*_*3*_. So when *C*_*1*_ is charged and discharged, the speeds on two paths are always different, resulting in a potential difference at the split gap of the SRRs. This is equivalent to an additional electrical excitation applied to the split gaps of the SRRs and is also similar to an external electromagnetic field excitation applied to the SRRs with an electric field polarized along the split gap. It is well known that the LC resonance mode in an SRR would be excited when the incident electric field is polarized along the split gap.

However, if the points connecting the bar resonator and the SRRs are located on the vertical center line of the SRRs, as shown in Fig. 6b, the two paths on the outer side and the inner side after the bifurcation are symmetrical. In this case, *R*_*1*_’ = *R*_*3*_’, *L*_*1*_’ = *L*_*3*_’. Therefore, the speed of charging and discharging along the two paths is always the same, and there is no potential difference.

**Fig. 7:**
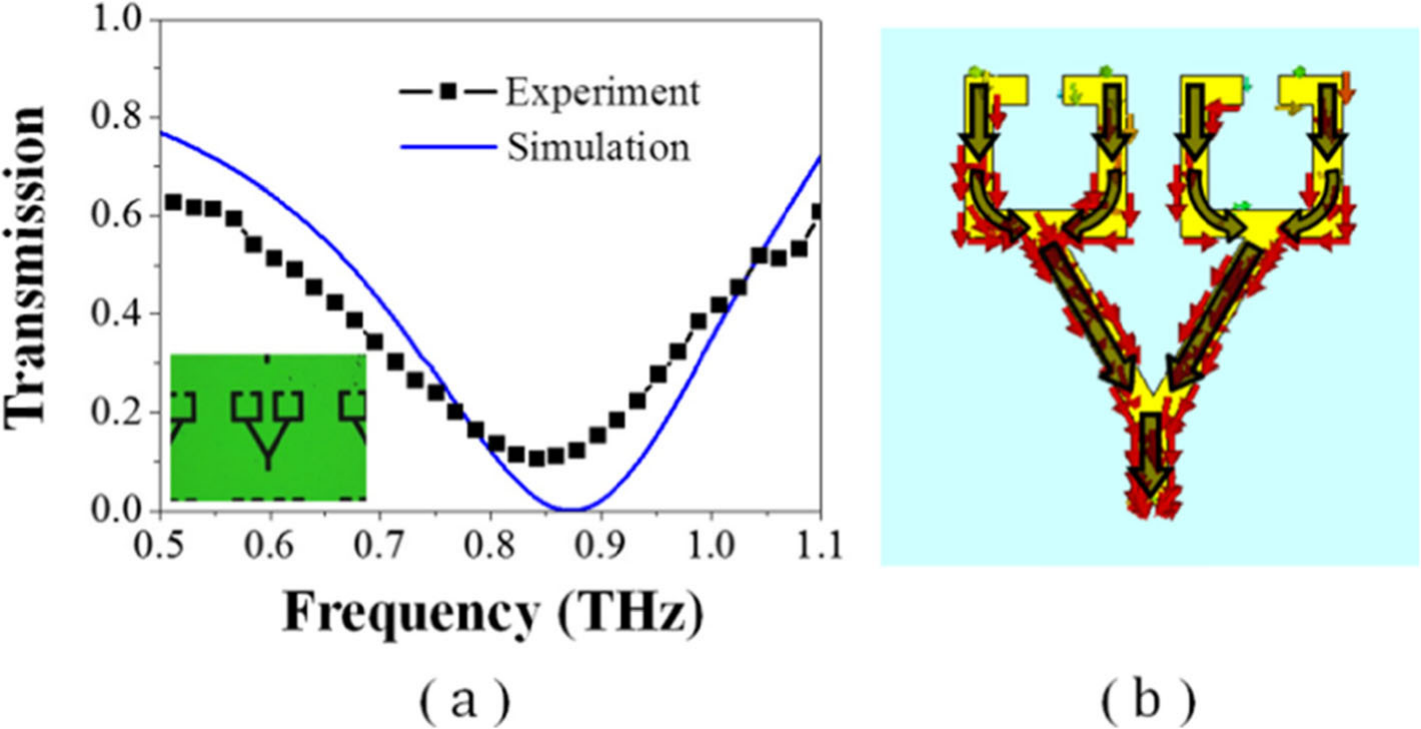
**a** Simulated and measured spectra of the conductively coupled terahertz metamaterial in which the junctions are located on the vertical center line of the SRRs. **b** Surface currents of the corresponding resonances

To verify the above conjecture, we designed and fabricated another metamaterial, in which the points connecting the bar resonator and the SRRs are located on the vertical center line of the SRRs. Thus, the length of the two conduction path, i.e., the currents flow along the metallic arm outside or inside the SRRs, can be the same. Figure 7a shows the simulated and measured spectra of this metamaterial. A microscopic image of the structure is also inserted next to it. Both the simulated and experimental results demonstrate that there is only resonance in this frequency range. Although the experimentally measured resonant frequency (approximately 0.85 THz) has some deviation from the simulated resonant frequency (approximately 0.87 THz), which mainly due to experimental errors, the measured curve and the simulation result are in good agreement. Figure 7b shows the surface current distribution when the resonance of this structure is induced, exhibiting a typical LSP resonance. Since the distances of the two conduction paths are the same, the amount of potential reduction through the two paths is also the same; there is no potential difference generated at the split gaps; therefore, the LC resonance and analog of EIT phenomenon cannot be formed.

As for the frequency of this LSP resonance (0.87 THz), it is higher than that of previous structure. It is because in the current structure, the surface current can flow through two conduction paths. This is equivalent to a parallel circuit where the resistance and the inductance are smaller than any one of the branches. This is the same as the effect of passing through a shorter conduction path. The conduction path becomes shorter, the resonance wavelength becomes smaller, and the resonance frequency becomes higher.

**Fig. 8:**
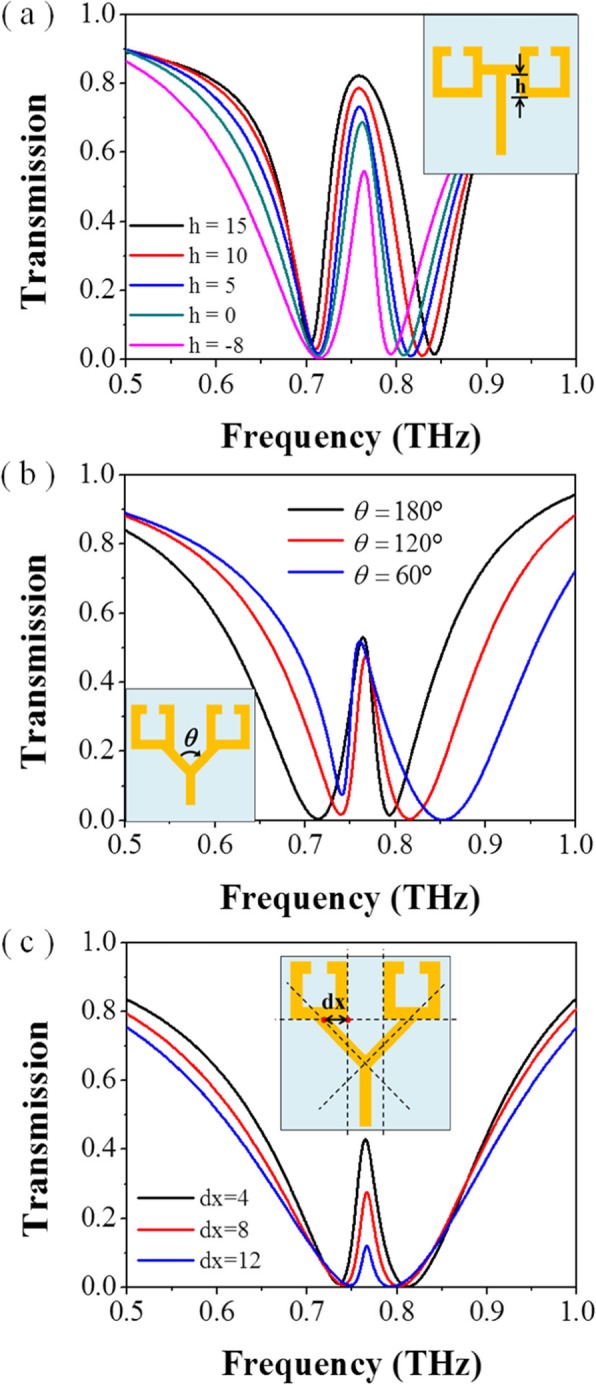
Simulated spectra of the conductively coupled terahertz EIT metamaterial **a** when the point connecting the bar resonator and the SRRs moves upward, **b** when the connecting bar in the middle is bent, **c** when the point connecting the bar resonator and the SRRs moves toward outward

We also simulated the influence of the asymmetry of the two conduction paths on the EIT phenomenon; the results are shown in Fig. 8. When the point connecting the bar resonator and the SRRs moves upward, as shown in Fig. 8a; the amplitude of the transmission peak increases accordingly.

In Fig. 8b, when the connecting bar in the middle is bent, which is to prepare for the movement of the connecting point toward outside, the frequency of absorption region of the EIT becomes higher with the increasing of the bending angle. When the bending angle increases, more parts of the conduction path are connected in parallel, i.e., conduction path becomes wider, which is the same as the effect of passing through a shorter conduction path. The conduction path becomes shorter, the resonance wavelength becomes smaller, and the resonance frequency becomes higher. This also explains why the resonant frequency in Fig. 7 is higher than that in Fig. 3. In Fig. 8c, when the joint point moves outward, the asymmetry decreases, the charge and discharge speeds of the *C*_*1*_ along the two paths tend to be the same; the potential difference becomes smaller, and the intensity of the dark mode gradually becomes weaker, leading to the decline of the transmission peak of EIT. This also reflects that the greater difference between the two paths along the SSR after bifurcation from the connection point, the stronger the effect of EIT.

**Fig. 9:**
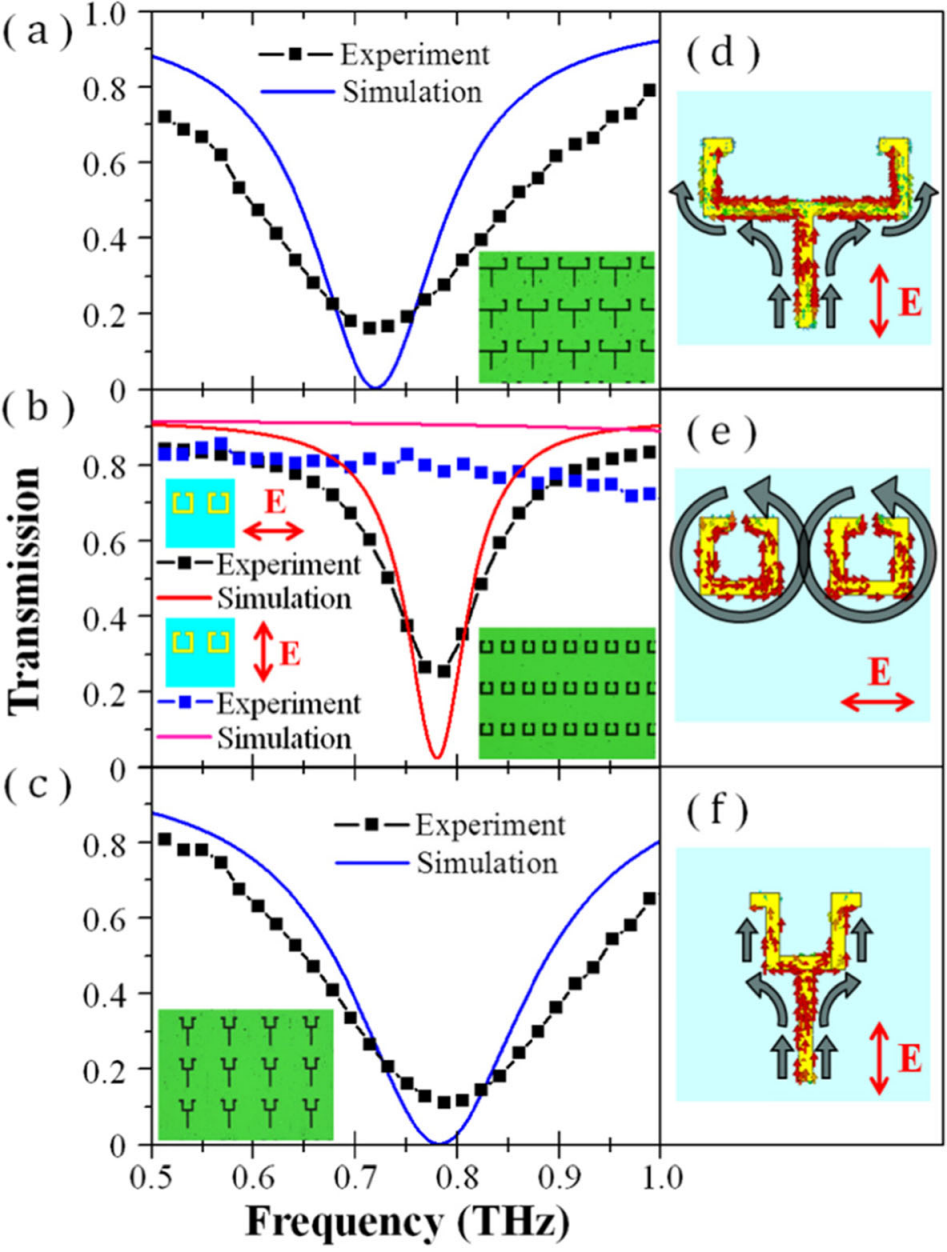
Simulated and measured spectra of different components of the conductively coupled terahertz EIT metamaterial: **a** the combined structure of the metal bar and the outer part of the SRRs, **b** SRR combination, **c** the combined structure of the metal bar and the inner part of the SRRs; The microscopic images of the fabricated components are also inserted in the corresponding spectra; **d-f** Surface currents of the corresponding resonances in **a-c**

We also separated the conductive metamaterial EIT structure and studied it separately. Figure 9 shows the simulated and measured spectra of the different components of the structure. As shown in Fig. 9a, the combined structure of the metal bar and the outer part of the SRRs produces a significant resonance at 0.72 THz when excited by an electric field polarized along the *y*-axis. Figure 9d shows the surface current distribution when the resonance of this structure is induced; this is similar to the distribution shown in Fig. 5a.

Although the direction is different, the overall trend in the surface current is considered the same, because the incident electromagnetic field is oscillating back and forth. Figure 9b shows the spectra of the SRR combination under incident light excitation with different polarization. When the electric field is polarized perpendicular to the direction of the split gap, no resonance occurs in the range of 0.5–1 THz, and the transmission remains at a high level. When the electric field is polarized parallel to the gaps of the SRRs, a resonance is generated at 0.78 THz. Figure 9e shows the surface current distribution when this resonance is excited. The surface current circulates back and forth on the surface of the SRRs, similar to the distribution shown in Fig. 5b. However, the flow direction of the two vortex surface currents, in Fig. 5b, is mirror-symmetric to the *y*-axis, whereas the vortex surface currents, in Fig. 5e, are in the same direction. This is because, in Fig. 9e, the resonances of the two SRRs are induced by the same electric field. Therefore, the direction of the vortex surface current is the same. However, in Fig. 5b, both the structure of the proposed metamaterial and the directions of the potential difference generated at the split gaps of the two SRRs are mirror-symmetric to the *y*-axis, thus making the excited surface current mirror-symmetric to the *y*-axis as well. The difference in the frequencies (0.76 THz vs. 0.78 THz) can be attributed to the fact that the vortex surface current in the conductive metamaterial is not strictly distributed only in the SRRs, and the elongation in the conduction path leads to an increase in the resonant wavelength, thereby making the frequency of the EIT peak (0.76 THz) slightly lower than the LC resonance frequency of the SRR combination (0.78 THz). As shown in Fig. 9c, the combined structure of the metal bar and the inner part of the SRRs produces a significant resonance at 0.79 THz under an electric field excited along the *y*-axis. Figure 9f shows the surface current distribution when the resonance of this structure is induced, exhibiting a typical LSP resonance. The resonances of the abovementioned components correspond to the conditions of the low-frequency transmission dip, the EIT transmission peak, and the high-frequency dip, respectively.

## Conclusion

In conclusion, we proposed a conductively coupled terahertz metallic EIT metamaterial, in which the bright and dark mode antennae are connected in the form of a fork-shaped structure. Aluminum foil, which is very cheap and often used in food package, is used to fabricate our metamaterials. Numerical and experimental analyses were conducted to analyze its mechanism. The surface current due to LSP resonance (bright mode) flows along different paths. Because of the asymmetry of the connection point with respect to the slit gap of the SRR, a potential difference is generated at the gaps of the SRRs. This is equivalent to an external electromagnetic field excitation with the electric field polarized along the slit gap. Thus, an LC resonance (dark mode) is induced, and the bright mode is suppressed, resulting in EIT. The proposed structure interacts via surface conducting currents. This may provide new ideas for the structural design of EIT metamaterials. Moreover, the process of fabricating microstructures on flexible substrates can provide a reference for producing flexible microstructures in the future.

## Data Availability

All data are fully available without restriction.

## Abbreviations

EIT: Electromagnetically induced transparency
LC: inductive-capacitive
SRRs: Split ring resonators
LSP: Localized surface plasmon
Q factor: Quality factor
PET: Polyethylene terephthalate
THz-TDS: Terahertz time domain spectroscopy
